# Enthalpic and Liquid-Phase Adsorption Study of Toluene–Cyclohexane and Toluene–Hexane Binary Systems on Modified Activated Carbons

**DOI:** 10.3390/molecules26102839

**Published:** 2021-05-11

**Authors:** Diana Hernández-Monje, Liliana Giraldo, Juan Carlos Moreno-Piraján

**Affiliations:** 1 Departamento de Química, Facultad de Ciencias, Universidad Nacional de Colombia, Sede Bogotá, Carrera 30 No 45-03, Bogotá 11001, Colombia; dichernandezmo@unal.edu.co (D.H.-M.); lgiraldogu@unal.edu.co (L.G.); 2 Departamento de Química, Facultad de Ciencias, Universidad de los Andes, Carrera 1 este No 18A-10, Bogotá 111711, Colombia

**Keywords:** liquid phase adsorption, enthalpy of immersion, activated carbon, toluene, cyclohexane, hexane

## Abstract

The liquid-phase adsorption of toluene in cyclohexane and hexane solutions on modified activated carbons was evaluated; the energy involved in the interaction between these solutions and the solids was determined by immersion enthalpies of pure solvents and their mixtures, and the contribution of the system constituents was calculated by differential enthalpies. The thermal treatment generated modifications that favored adsorption and interaction with the evaluated solutions, since it increased the textural parameters and the basic character of the samples. Cyclohexane could create greater competition with the adsorption sites compared to hexane, but it favored the increase in adsorption capacities (0.416 to 1.026 mmol g^−1^) and the interactions with the solid evaluated through the immersion enthalpies. The immersion enthalpies of pure solvents (−16.36 to −112.7 J g^−1^) and mixtures (−25.65 to −104.34 J g^−1^) had exothermic behaviors that were decreasing due to the possible displacement of solvent molecules when increasing the solute concentration in the mixtures. The differential enthalpies for toluene were negative (−18.63 to −2.14 J), mainly due to the π–π interaction with the solid, while those of the solvent–solid component tended to be positive values (−4.25 to 55.97 J) due to the displacement of the solvent molecules by those of toluene.

## 1. Introduction

Adsorption from liquid phase has been useful in processes such as removing compounds from effluents such as heavy metals, dyes, pharmaceuticals; oil recovery; purifying products (fuels); recovering valuable metals from leachates (Au, Ag, Cu); water treatment; separation of substances present in solutions or mixtures; and determination of surface area of certain materials, among others. To accomplish the liquid phase adsorption, there is an adsorbent (porous solid) and, in the liquid phase, there is generally a solute and a solvent. When they are put into contact, due to an imbalance of forces, the adsorbate is attracted by the porous material, causing the degrees of freedom to decrease. This continues until the equilibrium is reached; the adsorbent–adsorbate affinity defines the distribution of the solute and solvent in the solid and liquid phase [1,2].

**Figure 1:**
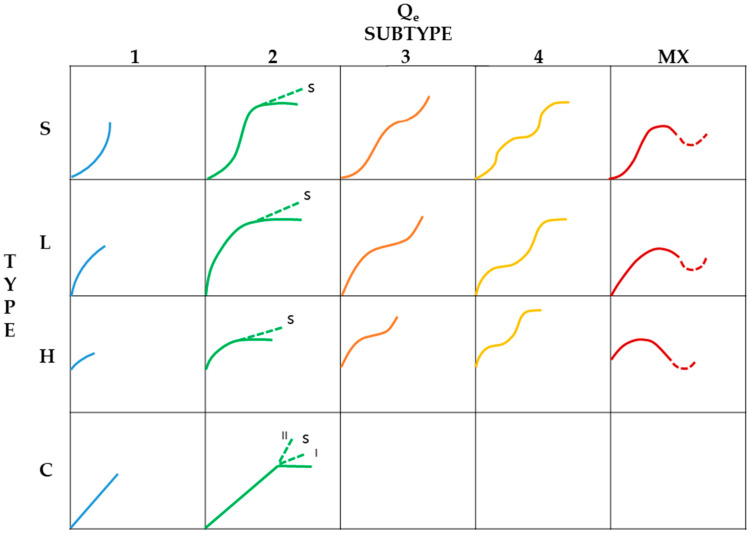
Isotherm classification for liquid phase proposed by Giles. Adapted and modified from [4].

In this kind of process, there is a competition between the solute and solvent where the “apparent adsorption” of the solute at the solid–liquid interface is frequently determined by quantifying the diminution of its concentration once it is put into contact with the porous material. According to an adsorption isotherm classification for liquid phase proposed by Giles et al. [3,4] (Figure 1: Adapted and modified from [4]) the curves of equilibrium are categorized in agreement with the shape of the initial slope: S, L, H and C; these shapes have subtypes: 1, 2, 3, 4, and mx which are related to the shapes of the upper sections and slope changes (s), where s is related to the slope, and I or II correspond to a sharp (I) or sharper (II) slope variation for the flat section of the graph for the C type isotherms.

S curves are convex at first, but later they become concave to the concentration axis (similar to type III or IV, according to the IUPAC classification [5]); L curves are concave to the axis of concentration (similar to type I isotherm, in line with the IUPAC classification [5]); H curves are similar to the L type, but they show higher affinity with the adsorbate, because the isotherm with the H type starts vertically oriented (similar to type Ia isotherm—IUPAC classification [5]); C curves possess linear behavior up to the maximum adsorption where an horizontal plateau is formed [1,6].

According to Limousin et al. [7] concave-shaped isotherms can be fitted to the Langmuir and Freundlich models which description is in Table 1:

Although adsorption in liquid phase has been widely used in the processes mentioned at the beginning of the introduction, it has been studied mainly for the aqueous phase [17,18,19,20], but little has been investigated about liquid phase adsorption for hydrocarbons when the solvent is another nonpolar organic substance [18], due to the research found is related to the usage of water as a solvent [17,18,19,20]; therefore, the study of systems of this type is interesting, since low-molecular-weight hydrocarbons are within the Volatile Organic Compounds (VOCs) which, due to their characteristics, can be in the environment in the gas and liquid phase.

Among these VOCs are toluene, cyclohexane and hexane, which are hydrocarbons that have been used in the manufacture of solvents, adhesives, oils, lacquers, cleaning products, paints, resins, coatings, inks, fats, synthetic fragrances, waxes, polymers for nylon and plastic soda bottles, colorants, rubber, drying glues and cement, polyurethanes, bitumen, nail cosmetics and pharmaceuticals [21,22,23,24,25,26,27]. These compounds have many uses, but it is interesting to study their adsorption because they can also generate adverse health effects such as harming the central nervous system; irritating the eyes, mucous membranes and skin; nausea, lightheadedness, dizziness, headaches, fainting, throat and respiratory tract disorders; vomiting; and lack of coordination; in the case of toluene, this is a carcinogenic and neurotoxic mutagenic agent [21,24,28,29,30,31,32,33,34].

Given the nonpolar nature of these contaminants, one of the most suitable adsorbents for their removal is activated carbon, since it also has a nonpolar nature. It is also used because it is a material that can generate a high content of microporosity and its surface chemistry can be modified in order to evaluate its affinity and adsorption capacity with compounds of this type.

According to the aforementioned outline of the topic, this research will work with activated carbon, and some modifications will be made to its porous structure and surface chemistry to evaluate the influence of these changes on the adsorption capacity of these modified solids. Since toluene is the most harmful of the three, it was chosen as the solute, while cyclohexane and hexane were chosen as solvents to evaluate the influence of size, molecular arrangement, and type of compound on the adsorption of toluene, since both they have six carbons in their structure, but one of them is an aliphatic with a chain closed and the other is a hydrocarbon with a chain opened with differences in the arrangement and size of their atoms.

To complement this study of adsorption of nonpolar solutions, an evaluation of the energy involved in the interaction of the toluene–hexane and toluene–cyclohexane mixtures with the activated carbons of different physicochemical characteristics will be carried out, through the thermodynamic parameter of enthalpy. A very interesting process that allows determining this variable is the immersion calorimetry since it establishes the energy that is released or required in the process of immersing the solid in the liquid phase of the system, allowing us to calculate the immersion enthalpy—which is related to the solid properties, such as the surface chemistry and porosity—and also the properties of the liquid as polarity, size, molecular arrangement and affinity to the carbonaceous solid [35,36].

For a closed system of solid–liquid interaction with one solute and one solvent at a determined pressure (*P*) and temperature (*T*), the variation of a thermodynamic parameter can be described by [37]:

(3) $$dX={\left(\frac{\partial X}{\partial P}\right)}_{T,\;n_{t}}dP+{\left(\frac{\partial X}{\partial T}\right)}_{P,\;n_{t}}dT+{\left(\frac{\partial X}{\partial n_{q}}\right)}_{P,T,\;n_{r}}dn_{q}+{\left(\frac{\partial X}{\partial n_{r}}\right)}_{P,T,\;n_{q}}dn_{r}$$

where *X* can be the immersion enthalpy (*H*), and

(4) $${\left(\frac{\partial X}{\partial n_{q}}\right)}_{P,T,\;n_{r}}={\left(\frac{\partial H}{\partial n_{q}}\right)}_{P,T,\;n_{r}}={\bar{H}}_{q}$$

(5) $${\left(\frac{\partial X}{\partial n_{r}}\right)}_{P,T,\;n_{q}}={\left(\frac{\partial H}{\partial n_{r}}\right)}_{P,T,\;n_{q}}={\bar{H}}_{r}$$

where ${\bar{H}}_{q}$ and ${\bar{H}}_{r}$ are the partial molar enthalpies for the components *q* and *r*; this process is performed at constant *P* and *T*, then the contribution of every component of the system to the enthalpy can be determined. 

As there is a difference among the partial molar enthalpy of any component, ${\bar{H}}_{q}$ and ${\bar{H}}_{r}$, and the pure component molar enthalpy, *H_q_***^∙^** and *H_r_***^∙^**, this difference allows us to determine the differential enthalpy change, $\Delta H_{De}$, for each component of the mixture, derived from the interaction between the solution and the adsorbent.

(6) $$\Delta H_{De}={\bar{H}}_{q}-H_{q}^{.}$$

The absolute values for the enthalpy are not able to be evaluated; however, the contribution of the component to the system can be determined. The differential enthalpy change can be expressed as follows:

(7) $$\Delta H_{De}={\left(\frac{\partial \Delta H_{exp}}{\partial n_{q}}\right)}_{P,T,n_{r}}$$

This parameter is interesting because it is useful to see the changes in the mixture and its properties when the quantities of the components are modified, since each of them contributes to the enthalpy of the mixture. This differential enthalpy and its variations could be expressed per mole; but if the changes in this parameter are calculated for a solution that interacts with an activated carbon, it is better to determine grams instead of moles of the component [38,39,40]. 

Additionally, for the determination of the solid differential enthalpy and solvent the Gibbs–Duhem equation is used:

(8) $$X_{solute}\left(\frac{\partial \Delta H_{De_{solute}}}{\partial X_{solute}}\right)+X_{solid-solvent}\left(\frac{\partial \Delta H_{De_{solid-solvent}}}{\partial X_{solute}}\right)=0$$

It allows us to determine the change of the partial quantities (related to the differential enthalpy) in regard to the composition of the system; this depends on T and P, but they are considered constant during the process. Through this mathematical relationship, the differential enthalpy variations for solvent and solid in the solid could be evaluated according to the solute fraction [38,39].

According to the above, this research will study the influence of the modifications in the physical and chemical characteristics of the adsorbent and the influence of each solvent, open chain hydrocarbon (hexane) and closed chain one (cyclohexane) in the toluene adsorption process; therefore, a study of a solid–liquid system will be made where the liquid phase is composed of two nonpolar compounds with affinity between them and, in turn, will be complemented with the evaluation of the interaction between the modified solids with toluene–hexane or toluene–cyclohexane mixtures expressed in terms of the thermodynamic parameter of the immersion enthalpy; on the other hand, the contribution of each component of the mixture will also be determined as the concentration of the solute increases.

So, the interesting aspect of this work is that nonpolar solutions are evaluated; this is a field that is not so commonly studied because it is more usual to find research that investigates aqueous systems [17,18,19,20]. 

In addition to the above, the calorimetry immersion technique offers added value because, besides being an unconventional technique, it allows us to calculate important thermodynamic parameters, such as the immersion enthalpy, both for pure solvents and for mixtures, going from the pure solvent immersion enthalpy (hexane or cyclohexane) to the enthalpy of immersion of the pure solute (toluene). This, in order to: (i) determine how the solid–liquid interactions change when the binary mixtures are put in contact with the adsorbent and (ii) analyze the contribution of the components of the solution and the activated carbon through the differential enthalpy change. Thus, the work of this manuscript is relevant since little has been studied in this aspect, because it has been evaluated for other materials such as graphite, zeolites [41,42,43] and some carbonaceous materials, but only for aqueous solutions [38,39].

## 2. Results and Discussion

Regarding the physical characterization for OAC (activated carbon prepared from coconut shell by physical activation subjected to an oxidization process at 358 K with 6 M HNO_3_ solution), OAC723 (oxidized sample “OAC” subjected at heat treatment up to 723 K under N_2_ atmosphere) and OAC1023 (oxidized sample “OAC” subjected at heat treatment up to 1023 K under N_2_ atmosphere); the solids do not show noticeable differences, but there is a trend, since the surface area (BET model, C positive) and the micropore volume (Dubinin–Radushkevich model) are: OAC 819 m^2^ g^−1^, 0.33 cm^3^ g^−1^ < OAC723: 873 m^2^ g^−1^, 0.35 cm^3^ g^−1^ < OAC1023: 908 m^2^ g^−1^, 0.37 cm^3^ g^−1^; this was also supported with the pore size distribution showed in another work [37], which demonstrated a pore volume for OAC<OAC723<OAC1023 with highly microporous behavior of the samples, where most of the pores had widths smaller than 20 Å. 

This was quite favorable, since the molecules of this research had sizes smaller than 20 Å which, when entering porous structures of these dimensions, will generate a high affinity with the adsorbent, since the adsorption potential is greater for micropores [44]. The fact that OAC was the sample with the smallest surface area and the smallest pore volume was due to modification with nitric acid which generated incorporation of surface groups (mostly acidic) that could decrease the textural parameters of the solid, while samples subjected to heat treatment slightly increase these characteristics, due to the possible transformation of some surface groups to CO and CO_2_. This is due to the modification temperatures 723 and 1023 K, which are also increasing the basicity of samples [37,45,46,47].

**Figure 2:**
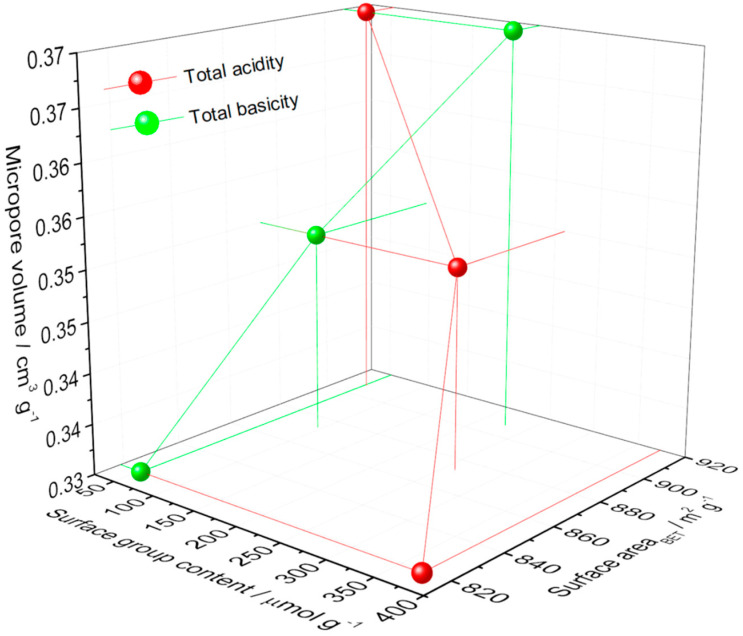
Relationship between the micropore volume and the surface area with the total acidity and basicity content of samples.

This corroborated the relationship between the micropore volume and the surface area with the total acidity and basicity content (the determination of acidity and basicity was shown in [48]) that can be seen in Figure 2. There was a directly proportional relationship between the textural parameters and basicity, but the relationship with total acidity was inverse, showing the dependence of these physical characteristics on surface chemistry. There were also greater changes in the values of acidity and basicity with respect to the surface area and the volume of the micropore, indicating a greater sensitivity of the surface groups to the thermal and chemical modifications carried out. The behavior of the graph may be due to the fact that oxidation with HNO_3_ increased the content of acid groups (mainly carboxylic [46]), possibly developed at the entrance of the pores, obstructing the entrance to certain regions of the porosity. This prevented the nitrogen from having access to these regions, decreasing the area and the volume to which the adsorbate had entry, while the total basicity increased the surface parameters because it was developed with the thermal treatment: when treating the sample at 723 K, acid groups such as carboxylic and lactonic were removed and, at 1023 K, the carboxylic, lactonic and phenolic groups [47]; this removal could enable entry to porosity that was not previously available, causing a greater amount of N_2_ to enter the porous solid, indicating a rise in micropore volume and surface area. 

With regard to the characteristics of the surface chemistry, Table 2 shows the ranges and peaks of the pK_a_ distributions of the activated carbons and their content in mmol g^−1^. In regard to pK_a_ < 7, the sample with ranges of lower values of pK_a_ and the peak with the highest acid character was OAC; later, the thermal modification generated a shift to the right of those ranges and peaks; this was quite interesting, since in pK_a_ between 2 and 4 the presence of carboxylic groups was detected. In pK_a_ between 4 and 7 the lactonic groups were detected [49,50]. Therefore, probably the oxidation with HNO_3_ favored the formation of these acid groups (on OAC), and then the acidity and content of these groups decreased as the temperature of thermal modification increased (sample OAC723); after, again, there was an increase in the functional groups content for OAC1023 but now at pK_a_ greater than 4, which could show that the surface chemistry changed, since at this treatment temperature the carboxylic groups (those with the highest acidic character evaluated) decomposed generating CO_2_; this removal was reflected in the range of the peak (3.6–4.8), which was very close to the limit of the pK_a_ for carboxylic acids (2 to 4); on the other hand, the presence of bands for all samples in the range between 4 and 7, but mostly for thermal modified samples (with shifted ranges and peaks to the right), made the presence of less acidic groups such as lactonics (pK_a_: 4–7 [50]) and pyrone-type groups (pKa: 5–6 [49]) more evident, since almost the entire range of the bands of samples was subjected to a temperature that corresponded to the pK_a_ of these functional groups, which might be present in higher concentration since, due to their thermal stability, they can still remain on the surface without transforming into CO_2_ up to temperatures close to 923 K [47].

For the pK_a_ > 7, the sample with the lowest content of groups of this type and less basic range was OAC since it contained 0.59 mmol g^−1^; when it was subjected to heat treatment, this amount practically doubled for OAC723 and OAC1023, and, for the latter, the content of the functional groups in the range 7–8 was also detected. The species that could be related to these ranges were the phenolic groups, since their pK_a_ > 7 [51,52], etheric cycles and ketones were separated by aromatic molecules whose pK_a_ were between 10 and 14 [49,53]; these rings have π electrons that also contributed to the basic character of activated carbons [49] and the content of these electrons could increase with increasing temperature, because the thermal treatment generated the removal of oxygenated groups, while the aromatic rings were more stable [47].

**Figure 3:**
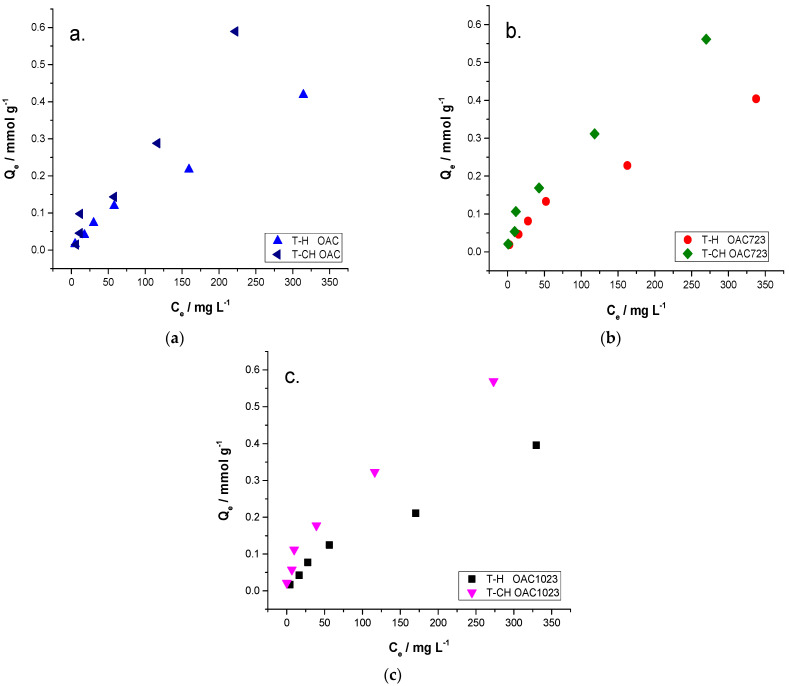
Experimental data of liquid-phase isotherms for the adsorption of solutions of toluene in hexane and cyclohexane for OAC (**a**), OAC723 (**b**) y OAC1023 (**c**).

After knowing information related to the textural parameters and the possible surface groups of the solids, the adsorption isotherms in liquid phase were carried out for the solutions of toluene in hexane and toluene in cyclohexane. In Figure 3, the experimental data of the isotherms in the liquid phase for the adsorption of the solutions for the samples OAC (a), OAC723 (b) and OAC1023 (c) were shown; they were separated in that way to better evidence their behavior. The three graphs show that the isotherms are the L type with concave form, according to Giles’ classification [1,3,4], and that there was greater adsorption when the solvent was cyclohexane and the trend of the slope was steeper for these systems; this could indicate that there was greater competition at high concentrations between cyclohexane and toluene for the entry of the pores, because they had close molecular dimensions in x and z for toluene and in x and y for cyclohexane (toluene: x = 6.625 Å, y = 4.012 Å, z = 8.252 Å; cyclohexane: x = 7.168 Å, y = 6.580 Å, z = 4.982 Å [54]), having similar probabilities of entering to the pores if they were oriented in these ways. However, if hexane was the solvent, there was a lower inclination towards the end points, which could be due to the fact that it had some much larger dimensions in x (x = 10.344 Å, y = 4.536 Å, z = 4.014 Å [54]) compared to any dimension of toluene in any orientation. Therefore, if it is located in this way, it will hardly be able to enter, causing reduced competition for adsorption sites, which made it easier to reach a plateau due to the monolayer formation of the solute [1].

**Figure 4:**
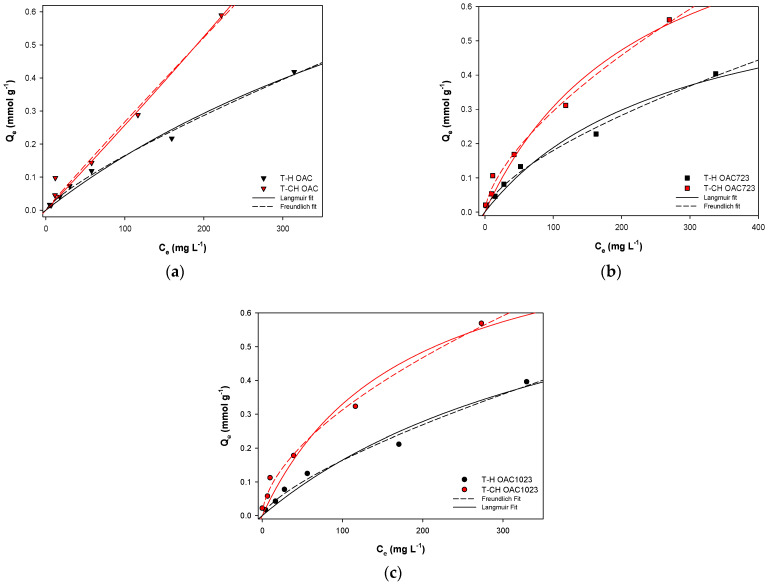
Liquid-phase isotherms for the adsorption of solutions of toluene in hexane (T-H) and cyclohexane (T-CH) for OAC (**a**), OAC723 (**b**) y OAC1023 (**c**) adjusted to Freundlich and Langmuir models.

Due to the fact that isotherms showed a concave form, they were adjusted to the models of Langmuir and Freundlich, according to the suggestion of Limousin et al. [7]; the results are shown in Figure 4 and Table 3. According to the adsorbed amounts, they were within the ranges found in other studies (only one was found where toluene–cyclohexane adsorption was studied, but in polymeric resins) [55,56,57,58,59] and if toluene solutions were compared in hexane and cyclohexane, adsorption was higher when the solvent was cyclohexane for all samples; as for activated carbons, the adsorbed amount was much lower for the oxidized sample without heat treatment and increased for samples that were exposed to heat treatment. The same occurred with Freundlich’s constant, which was also associated with the adsorption capacity; this parameter presented the lowest values for the OAC sample and increased for OAC723 and OAC1023 and the highest values were for the toluene–cyclohexane systems. On the other hand, the parameter 1/n was less than 1 in all the samples, indicating that they were activated carbons with heterogeneous surfaces [8,11,12,13,14,15,16].

Regarding the correlation constants, the solutions where hexane was the solvent fitted better to Langmuir model, while those where the solvent was cyclohexane fitted better to Freundlich’s. This might be due to the energetic heterogeneity of the surface, given the diverse surface chemistry of the samples described above that generated adsorption sites of different nature and energy, and also the possibility of formation of multilayer that both the solvent and the solute had. 

The mentioned behavior was an indication of what was described above, where it was said that, since there was less competition between toluene and hexane due to their molecular dimensions, there was a greater possibility of monolayer filling for the solute, which would decrease the slope of the isotherm (approaching to Langmuir-type isotherm shape). 

On the other hand, the similar dimensions of toluene and cyclohexane generated greater competition for adsorption sites, mainly at higher concentrations because there were fewer adsorption sites, making it a little more difficult for toluene to generate a monolayer on the solid surface. This caused that the slope of the isotherm did not reach a plateau and it was more evident in the OAC sample because, as it contained a smaller volume of micropore, there was greater competition for entering the porous system, showing a greater slope than the other samples.

What could happen is that, according to references, at low concentrations, all these molecules were oriented perpendicular to the surface [60,61,62]. Additionally, in pore sizes less than 10 Å, both toluene and cyclohexane did not have any restriction regarding the type of orientation; then, at sizes greater than 12 Å, hexane had no longer restrictions in its orientation, toluene could form two layers in parallel, while cyclohexane was oriented vertically or inclined to the surface; from 14 Å, the orientation of toluene changed and more layers could be formed to place one molecule in parallel and another perpendicular, while cyclohexane, by forming more layers, made hexagonal lattice packaging [60,61,62]. This type of organization of the hydrocarbons in the porous structure is what could have generated the behaviors of the adsorption isotherms.

**Figure 5:**
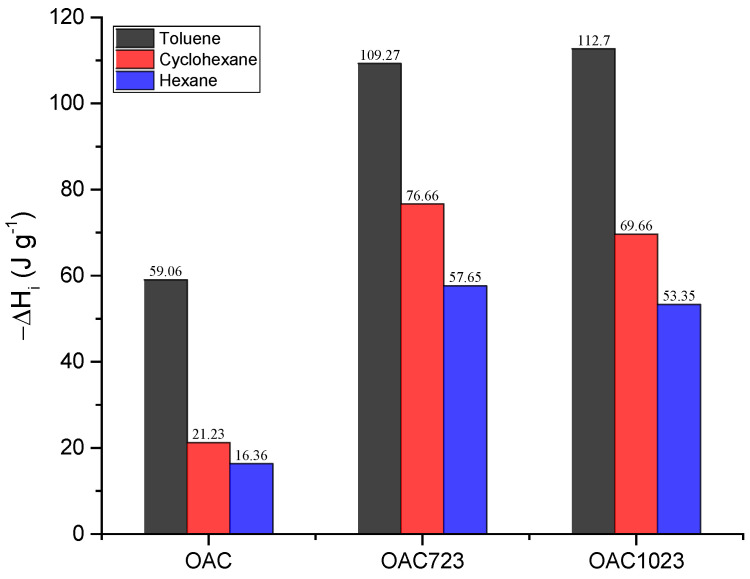
Immersion enthalpies of samples into toluene, cyclohexane and hexane.

After characterizing the isotherms in the liquid phase, the study was complemented with the calculation of the immersion enthalpies of the solids in the pure solvents and their mixtures. The immersion enthalpy changes of the pure solvents are in Figure 5. 

Since the immersion enthalpy was related to the energy released or required in the immersion of a porous solid into a liquid, all processes were exothermic (ΔH < 0: in the plot −ΔH > 0), as shown in Figure 5, where the intensity of the interaction is toluene>cyclohexane >hexane for all samples; this could occur because toluene is an aromatic molecule, so it had delocalized π electrons that interacted with the structure of activated carbon, increasing the immersion enthalpy values, while cyclohexane and hexane were aliphatic compounds but differed in their dielectric constant (hexane: 1.89, cyclohexane 2.02 [63,64]). This indicated that, despite being nonpolar compounds, the distribution of the cyclohexane electron cloud is more sensitive to an electric field [65]. As there was a force field in the solid present in the micropores, it caused the electronic cloud of the closed-chain compound to be distorted more easily, increasing the dispersive attractions with respect to the open-chain aliphatic, which would make the intensity of the interaction higher for cyclohexane and much lower for hexane, as shown by the magnitudes of the enthalpies. Likewise, it was seen that the least exothermic immersions were those of the sample modified with nitric acid, which occurred because the presence of acidic groups, could reduce the interaction with nonpolar compounds. Furthermore, in another work [48], the hydrophobic factors for these samples were shown, and it was demonstrated that the oxidized sample without heat treatment presented the lowest hydrophobic factor of the evaluated solids (OAC: 1.43; OAC723: 2.02; OAC1023: 3.44), meaning that there was less affinity for hydrocarbons.

**Figure 6:**
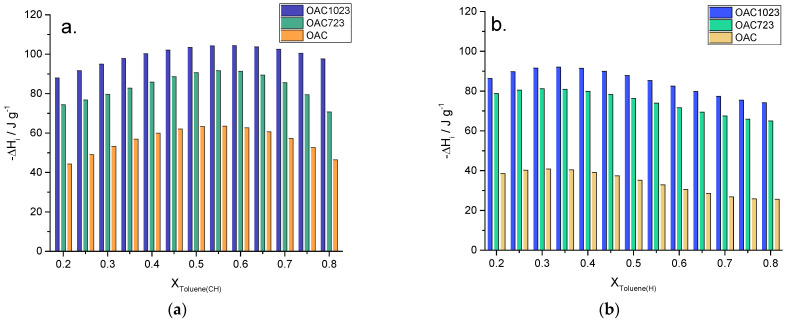
Immersion enthalpies of samples into toluene–cyclohexane (**a**) and toluene–hexane (**b**) mixtures.

After evaluating the enthalpies in the pure solvents, Figure 6 shows the immersion enthalpies of solids into the mixtures of toluene–cyclohexane (a) and toluene–hexane (b).

Regarding the samples, it was observed how the interaction was OAC<OAC723<OAC1023, because the increase in temperature generated the removal of groups such as carboxylics and this created unsaturations on the surface, which increased the basic character of the solids. This basicity was highly related to delocalized π electrons located at the edges of the graphene layers [49]; due to the fact that toluene was also a compound with these unsaturations, the interaction with these types of compounds increased with the basicity of solids; in addition, the samples treated at 723 and 1023 K had greater pore volume and surface area, generating the possibility that more molecules could have contact with the solid and therefore there was more energy involved in the process.

With respect to the mixtures, they showed differentiated behavior between them; this may be due to the size and orientation of the solvents. As cyclohexane was the solvent with smaller sizes than 10 Å in all its dimensions, if it entered the porous structure it would allow more toluene molecules to enter to the system with respect to hexane, which meant that as the molar fraction increased, the immersion enthalpy increased too, as shown in Figure 6, since the enthalpy increased up to a molar fraction of 0.55 for cyclohexane and only approximately to 0.3 for hexane, starting to decrease slightly; then, when the porous structure became saturated, a displacement of the cyclohexane or hexane molecules by toluene molecules occurred, which implied an endothermic process that was reflected in the decrease in the immersion enthalpy values for the higher values of molar fraction of toluene.

**Figure 7:**
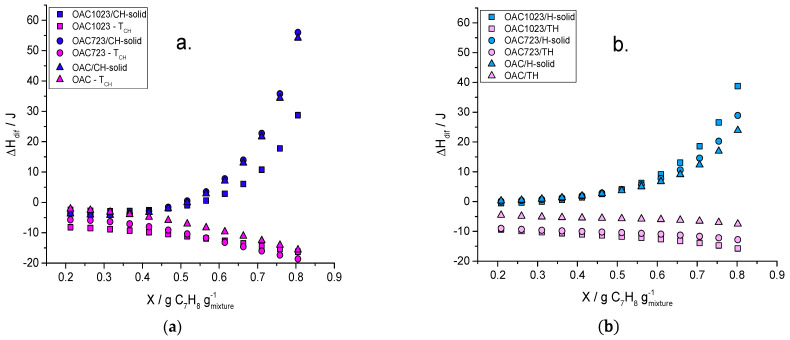
Differential enthalpies of toluene and solvent–solid component: cyclohexane–solid (**a**) and hexane–solid (**b**) in function of the mass fraction of C_7_H_8._

Finally, Figure 7 shows the changes in the differential enthalpies of toluene and cyclohexane–solid (a) and hexane–solid (b) component as function of the mass fraction of C_7_H_8_ (g_toluene_/g_mixture+solid_).

In Figure 7, the solute exhibited an exothermic behavior throughout the entire range of mixtures; this indicated that the intensity of the interaction increased with the concentration. This might be due to the fact that, at the beginning, there was an entrance of C_7_H_8_ into the porous structure and then, the solvent molecules displacement occurred. Therefore, although energy was required to remove these molecules, once toluene entered to that adsorption site, its interaction was greater than that of cyclohexane or hexane, since its dielectric constant was higher (2.38 [63]); in turn, toluene could also arrange in multilayers, which would imply greater interaction in the system, since the electron density would increase due to the adsorbate, generating more CH–π and π–π interactions with the surface [61].

This was corroborated by the outcome of the samples, since the enthalpy was lower for the sample modified with nitric acid in both systems and increased for the samples thermally modified which had higher basicity, and, therefore (as previously discussed), higher content of delocalized π electrons. Furthermore, the interaction with toluene had an additional contribution due to the methyl that was the electron donor, which increased the electronegativity of the aromatic ring, creating interactions of greater intensity with the nucleophilic part of the basal plane of the activated carbon [17,64,65,66].

With respect to the solvent–solid component, an contrasting behavior was evident, since as the mass fraction of toluene increased, the process became endothermic, raising its magnitude towards that direction; this could imply that, from these amounts of toluene, the process of displacement of the solvent molecules by those of the solute started, requiring energy to achieve it. It was also seen that the values are more positive for cyclohexane than for hexane. This could be due to the fact that the interaction with the closed chain aliphatic could be stronger than with hexane because more molecules could have been adsorbed (cyclohexane can form a multilayer from 12 Å [62]) requiring more energy to remove them or because the interaction with the attractive field of the micropores was greater due to its dielectric constant when it is compared to that of the open-chain aliphatic.

## 3. Materials and Methods

### 3.1. Samples

**Figure 8:**
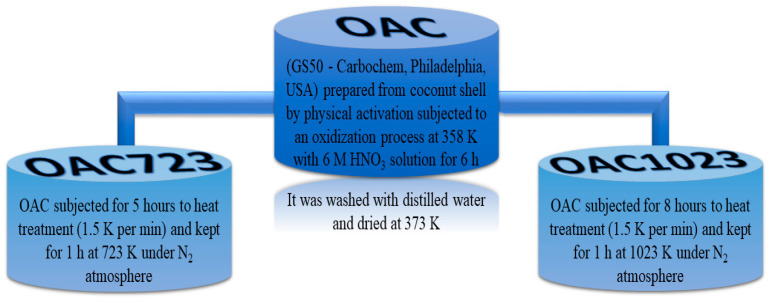
Scheme of the conditions and modifications of the samples [48].

The conditions and modifications of the samples are shown in Figure 8:

### 3.2. Physical Characterization of Samples

To determine surface area, micropore volume and cumulative pore volume N_2_ gas-phase isotherms were performed in an Autosorb 3B equipment from Quantachrome. Approximately 80 mg of activated carbon were degassed at 10^−5^ mbar at 473 K during 24 h. After, the sample was placed in station for the adsorption, adjusting the temperature at 77 K, and N_2_ was supplied at relative pressures for the adsorption branch from 7 × 10^−5^ up to 0.99 and the desorption was performed up to a relative pressure of 0.3 (equilibrium time: 3 min) [48].

### 3.3. Chemical Characterization of Samples

Different surface groups according to their pK_a_ value were determined through potentiometric titrations which were carried out by mixing 100 mg of solid (previously dried −378 K, 24 h) with standardized solutions of NaNO_3_ (50 mL, 0.01 mol L^−1^) and HCl (1 mL, 0.1 mol L^−1^) under stirring and nitrogen atmosphere for 24 h. Then, a blank (HCl, 0.1 mol L^−1^) was titrated with NaOH 0.1 mol L^−1^ in a Metrohm AG automatic titrator, ref. 905 Titrando - tiamo V2.2 software [67,68], after the system of interest under N_2_ (hydroxide flow: 0.01 mL every four minutes). The titration curves were then transformed to proton binding curves, data were smoothed, the proton binding curve was deconvolved and the pK_a_ spectrum was obtained by SAIEUS method [68,69,70,71]; then, this spectrum was decomposed into a sum of Gaussian functions [72].

### 3.4. Hydrocarbons Adsorption from Liquid Phase

The solid (100 mg) was mixed with 10 mL of toluene–hexane or toluene–cyclohexane solutions of different concentrations (20–500 mg L^−1^) at 273 K (solute: toluene; solvents: hexane and cyclohexane) for the time required to achieve the equilibrium. Then, the final concentration of each solution was evaluated with a gas chromatograph (GC-2010).

### 3.5. Calorimetric Evaluation of the Immersion Enthalpy for Hydrocarbons and Their Mixtures

**Figure 9:**
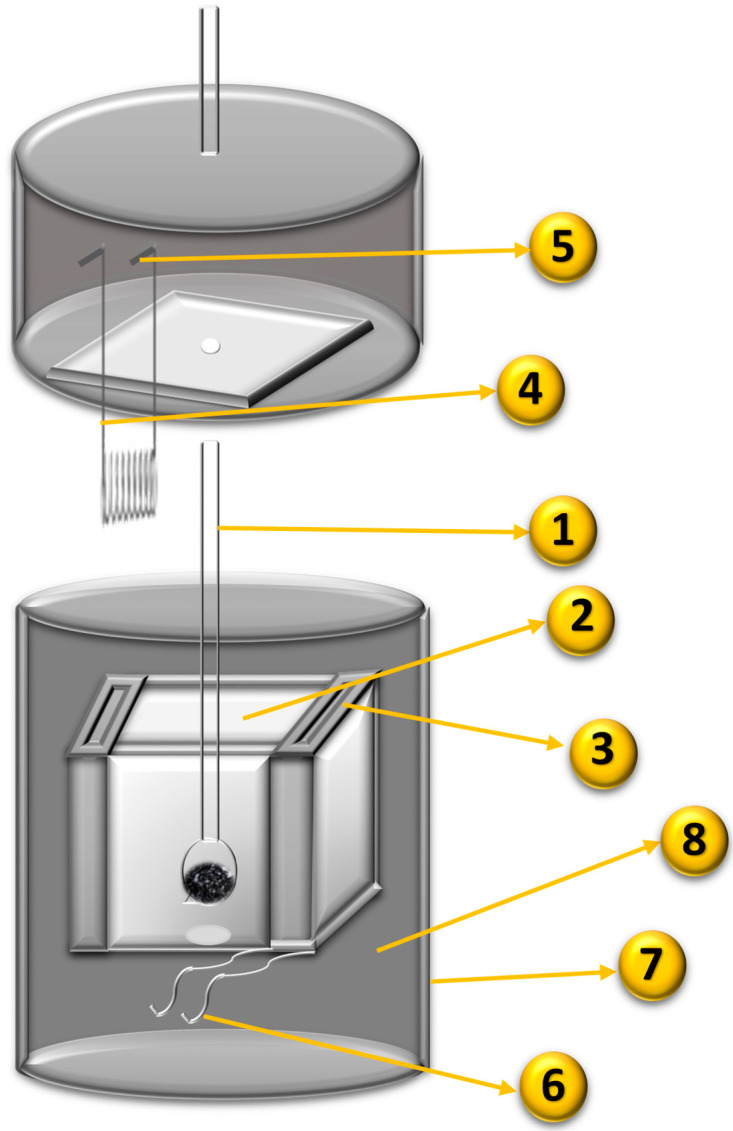
Scheme of the heat conduction microcalorimeter: 1. Glass bulb with fragile brittle end; 2. Cell for the liquid of immersion; 3. Sensors; 4. Resistance; 5. Output connection to power source; 6. Output connection to the multimeter; 7. Cover for insulation; 8. Heat dissipator. Adapted and modified from [73], p. 171.

The evaluation of the enthalpy of immersion was performed in a heat conduction microcalorimeter (Figure 9) [73]. A total of 100 mg of activated carbon was deposited in the glass bulb (1, in Figure 9) that had a small and fragile brittle end, and then this cell was assembled to the device; on the other hand, 10 milliliters of the liquid of interest was placed in a cell made of stainless steel (2, in Figure 9: Adapted and modified from [73]) that was also inserted into the calorimeter. Then, the change in potential over time measured by the sensors (3, in Figure 9) was captured until the system reached equilibrium (a straight line was observed in the calorimetric curve); later, downward pressure was applied to the glass cell to break the tip for performing the immersion of the carbonaceous material in the liquid phase; this created a change in the potential that was captured until it reached equilibrium again, so the calibration was made with a resistance of 100 Ω (4, in Figure 9) to determine the constant of the calorimeter [37].

The liquids of immersion were: toluene, cyclohexane and hexane each separately and then mixtures of toluene–cyclohexane (T-CH) and toluene–hexane (T-H) with molar fractions from 0.2 to 0.8, for example: T-CH(0.2) means 0.2 mol C_7_H_8_/0.2 mol C_7_H_8_ + 0.8 mol C_6_H_12_ or T-H(0.4) means 0.4 mol C_7_H_8_/0.4 mol C_7_H_8_ + 0.6 mol C_6_H_14_ [37].

## 4. Conclusions

Toluene adsorption capacities from the liquid phase using hexane and cyclohexane as solvents were between 0.416 and 1.026 mmol g^−1^, where the highest values were obtained when the solvent was cyclohexane and if the sample had a higher surface area, micropore content and higher basicity (with respect to the ranges and groups content according to the pK_a_); there was possibly greater competition for the adsorption sites with cyclohexane than with hexane, given the characteristics of size and molecular arrangement within the porous structure.

The enthalpies of immersion of the pure solvents were exothermic for all samples and all solutes with values between −16.36 and −112.7 J g^−1^, where the highest values were for toluene and the samples subjected to heat treatment due to the π–π interaction between the solid and the aromatic compound.

The enthalpies for the mixtures had exothermic values in a range between −25.65 and −92.05 J g^−1^ for toluene–hexane and between −44.24 and −104.34 J g^−1^ for toluene–cyclohexane, where the interaction was greater, with OAC1023 for being the most hydrophobic sample and with cyclohexane because it had smaller sizes than 10 Å in all its dimensions, allowing a greater entry of solute to be achieved than the hexane that had larger dimensions in x.

The differential enthalpies showed values for toluene between −18.63 and −2.14 J, being exothermic throughout all the concentration because of CH–π and π–π interactions with the surface and the methyl radical that was the electron donor. This created interactions with the nucleophilic part of the basal plane of the activated carbon; this interaction became higher in the samples with higher basicity due to their higher content of delocalized π electrons. For the solvent–solid component, the range was from −4.25 to 55.97 J, being more endothermic for cyclohexane because it could form multilayer and also greater interactions with the attractive field of micropores than hexane due to its dielectric constant, causing more energy to be required to its displacement.

## Figures and Tables

**Table 1:** Description of Langmuir and Freundlich models [8,9,10,11,12,13,14,15,16].

Model	Langmuir [8,9,10,11,12]	Freundlich [8,11,12,13,14,15,16]
Statements	The surface has finite active centers with the equal probability to adsorb a single molecule. The energy of adsorption in monolayer is the highest. More pressure is needed for formation of successive layers.	Empirical model for reversible and non-ideal adsorption. Different energies of adsorption for the active sites. Heterogeneous surface where multilayer can be formed.
Equation	(1) $n=n_{m}\frac{bC_{e}}{\left(1+bC_{e}\right)}$ *n* (mmol g^−1^): adsorbed amount at an equilibrium concentration n_m_ (mmol g^−1^): maximum adsorption capacity b (L mmol^−1^): Langmuir constant associated with adsorption energy C_e_ (mmol L^−1^): Concentration at equilibrium	(2) $n=K_{f}C_{e}^{1/n}$ *n* (mmol g^−1^): adsorbed amount at an equilibrium concentration K_f_ (mmol g^−1^ * mmol L^−1^): Freundlich constant related to adsorption capacity C_e_ (mmol L^−1^): Concentration at equilibrium 1/n: related to surface heterogeneity

**Table 2:** Ranges and peaks of the pK_a_ distributions of the activated carbons and their content in mmol g^−1^.

Samples			OAC	OAC723	OAC1023
pK_a_ < 7	pK_a_ 2–4	Range	2.7–3.5	3.2–4.0	3.6–4.8
		Peak	3.15	3.58	4.16
		Content (mmol g^−1^)	0.08	0.02	0.07
	pK_a_ 4–7	Range	4.7–5.6	4.8–5.8	5.4–6.4
		Peak	5.14	5.29	5.95
		Content (mmol g^−1^)	0.03	0.04	0.04
pK_a_ > 7	pK_a_ 7–8	Range			7.0–8.0
		Peak			7.43
		Content (mmol g^−1^)			0.05
	pK_a_ 10–12	Range	10.2–11.2	10.6–11.6	10.8–11.8
		Peak	10.7	11.09	11.27
		Content (mmol g^−1^)	0.59	1.02	1.01

**Table 3:** Fitting of liquid-phase isotherms to Langmuir and Freundlich model.

Model↓	System →	Toluene–Hexane			Toluene–Cyclohexane		
		OAC	OAC723	OAC1023	OAC	OAC723	OAC1023
Langmuir	n_m_ (mmol g^−1^)	0.416	0.714	0.903	0.724	1.026	0.910
	b (L mmol^−1^)	0.007	0.004	0.002	0.005	0.004	0.006
	R^2^	0.986	0.977	0.970	0.915	0.974	0.967
Freundlich	K_f_ (mmol g^−1^) (mmol L^−1^)	0.004	0.009	0.006	0.003	0.016	0.022
	1/n	0.795	0.652	0.715	0.968	0.636	0.580
	R^2^	0.993	0.993	0.986	0.979	0.992	0.992

## Data Availability

The data presented in this study are available on request from the corresponding author.

## Sample Availability

Samples of the solids are available from the authors.
